# Medical Student Intentions to Move Abroad: A UK-Based Realist Evaluation

**DOI:** 10.5334/pme.1170

**Published:** 2024-02-21

**Authors:** Elizabeth Mcculloch, Dominic W. Proctor, Karen Mattick

**Affiliations:** 1 Torbay and South Devon NHS Trust, Torbay, UK; 2 University of Exeter Medical School, St Luke’s Campus, Heavitree Road, Exeter, EX1 2LU, UK; 3 Royal Free London NHS Foundation Trust, London, UK; 4 University of Exeter Medical School, Exeter, UK

## Abstract

**Introduction:**

Medical students moving abroad after qualification may contribute to domestic healthcare workforce shortages. Greater insights into how medical students make decisions about moving abroad may improve post-qualification retention. The aim was to develop a programme theory explaining medical students’ intentions to move abroad or not.

**Methods:**

In Phase 1 the initial programme theory was generated from a literature review. In Phase 2, the theory was developed through 30 realist interviews with medical students from a medical school in the United Kingdom. In Phase 3 the final programme theory was used to produce recommendations for stakeholders.

**Results:**

The findings highlight the complex decision-making that medical students undertake when deciding whether to move abroad. We identified five contexts and six mechanisms leading to two outcomes (*intention to move abroad and no intention to move abroad*).

**Conclusions:**

This realist evaluation has demonstrated how contexts and mechanisms may interact to enable specific outcomes. These insights have allowed evidence-based recommendations to be made with a view to retaining graduates, including protected time within medical curricula to experience other healthcare systems, improved availability of domestic postgraduate posts providing domestic career certainty and stronger domestic-based social support networks for graduates.

## Introduction

Retention of healthcare staff is an international challenge of critical importance [1,2]. While the most substantial workforce shortages are reported in low-income nations [3], pressures are mounting across high-income nations. For example, in 2023 the United Kingdom (UK) National Health Service (NHS) reported a vacancy rate of 8.0% and an overall medical vacancy rate of 5.8%, not accounting for substantive vacancies filled by temporary staff [4]. These deficits may result in poorer patient outcomes, longer waiting lists and decreased staff satisfaction.

High rates of doctor migration are a significant contributor to workforce shortages [5,6]. As a result, migratory intentions and outcomes in qualified doctors have been researched extensively with the dominant theory being the push-pull model, which focuses on disparities in conditions and contexts to explain medical migration patterns [7]. Other models include person-environment fit theory [8] that recognises the role of different occupational interests in social career decision-making. It is possible that a combination of career decision-making and migratory theories interact to underpin individual decisions about where to work.

Doctor migration in high-income nations occurs most frequently in early-career clinicians under the age of 30 [9] with reported push-pull factors including financial considerations, the pursuit of better working conditions and positive work experiences (such as working in a preferred speciality) [9,10,11,12,13]. What remains unclear is whether these factors initiate a desire to migrate among qualified clinicians, or whether they are merely catalysing a pre-existing desire to work abroad that may have been formed as a medical student. Medical school experiences have been shown to significantly impact medical students’ career decisions through the influence of qualified clinicians [14]. Data from the Organisation for Economic Co-operation and Development also suggest that medical student international mobility among high-income nations is increasing, with only a quarter of UK-based international medical students renewing their visas after qualification [15]. Therefore, it remains possible that the desire to move abroad pre-dates medical qualification in certain individuals. Medical student perspectives on migration have been studied previously in other countries (Austria, Croatia, Ghana, Spain and Uganda) [16,17,18,19,20,21], with students highlighting perceived poor working conditions and a lack of future training opportunities in their home countries as primary motivators to move abroad.

However, these studies have not addressed the influence of specific educational contexts, nor have they identified means of improving student perspectives and the resulting impact on student opinions. There is also a relative paucity of data from high income countries and to our knowledge, no formal investigation into intentions to move abroad among UK medical students has been undertaken. An improved understanding of the medical student perspective may help design and implement effective interventions to improve retention of domestically trained medical students.

## Methods

The aim of this study was to generate a theory explaining why UK medical students decide to move abroad or not, in order to produce recommendations to help stakeholders improve graduate retention. This was further divided into three research phases with distinct objectives.

1. Identify existing contexts and decision-making processes that influence where medical students choose to work, to form an initial programme theory based on peer-reviewed literature.
2. Develop the programme theory through realist interviews with medical students, to identify which contexts and mechanisms are most likely to lead to which outcomes.
3. Produce recommendations to generate positive educational outcomes, for example improved retention of medical students.

### Study design

Realist research expands our understanding of why events occur by connecting contexts (circumstances) to mechanisms (reasons underpinning opinions) and outcomes (what happens), uncovering ‘what works best, for whom, under what circumstances and how’ [22]. Allowing for evaluation in complex environments, they consider the substantial role of social context in theory implementation [23].

Realist research starts with an initial programme theory, beginning to explore the relationships between contexts, mechanisms, and outcomes. Although this could be derived in a variety of ways, [24] it is usually produced through a different approach to the subsequent theory development. This initial programme theory does not have to be realist in nature [24]. It is the basis of presentation for a relevant population to iteratively validate, falsify, or modify reflecting their experiences [25]. Ultimately, researchers aim to produce a final programme theory with confidence that it represents their population’s experience as accurately as possible. The programme theory usually depicts how and why an intervention leads to outcomes; however Pawson and Tilley explain that uncovering why a behaviour occurs and how one acts can in itself be a programme [26].

The realist evaluation’s strength of uncovering insights in specific social settings can help to understand a specific population. Given the wide literature regarding the circumstances (contexts) in which medical students from many international backgrounds decide whether to move abroad (outcomes), a realist evaluation is well-equipped to uncover medical students’ reasoning (mechanisms) whilst they are in a specific context and analyse connections within this complex topic.

For this research we used the RAMESES II guidelines [24] and Manzano’s approach to realist interviewing [27]. Realist interviews were chosen since the conversational and participant-led nature had the potential to unearth more data than other methodological approaches, such as surveys.

### Definitions

Distinguishing between contexts, mechanisms and outcomes can be challenging [28]. Therefore, for this research, Pawson and Tilley’s definitions [26] have been applied (Box 1). Contexts, mechanisms and outcomes are connected and conveyed as context-mechanism-outcome configurations (CMOcs), the ‘analytical unit of realist evaluation’ [24]. Building on Pawson and Tilley’s context definition, Greenhalgh and Manzano [29] highlighted how contexts may be defined as ‘A thing that triggers’ including the fixed property of individuals that has straightforward impacts on outcomes. Furthermore, Coldwell outlined that contexts may be static in nature [30]. Given these further definitions; demographic contexts were included in the initial programme theory.

### Phase 1: Generating an Initial Programme Theory

As is typical in realist research, we conducted a rapid review of literature [31] aiming to assess existing peer-reviewed literature of medical students’ decision making regarding moving abroad, to create the initial programme theory. The following databases were searched from first records until November 2021: Ovid MEDLINE and EMBASE, ERIC (Educational Resources Information Center) and the Cochrane Database of Systematic Reviews. Keywords were searched using combinations of populations and outcomes to discover relevant literature (Appendix 1, Table 1). If potential contexts were identified, these were added to the search strategy to identify related mechanisms. Forward and backward citation searching identified additional literature. Relevant records (identified on screening of title and abstract) were read in full, with extraction of contexts and mechanisms regarding medical students’ intensions to move abroad or not.

**Table 1:** Definitions used in this UK-based realist evaluation.


CONCEPT	DEFINITION

Contexts	Contexts can often be changed, and it is only when a person is in that context that it activates a mechanism to produce an outcome.

Mechanisms	Mechanisms are the internal reasonings or responses that trigger a person to or not do something (outcome) when they are in a set of certain circumstances (contexts). Mechanisms are often hidden, commonly depicting a person’s feelings or perceptions about their contexts.

Outcomes	Outcomes encompass the intended or unintended consequences resulting from mechanism activation in different contexts.


All contexts, mechanisms and outcomes identified were included in the initial programme theory. If multiple literature sources identified connections between these, they were included in the programme theory. Further connections were added upon discussion by the research team based on real-world experience.

### Phase 2: Realist interviews

The realist interviews aimed to iteratively develop the programme theory. Medical students from a university in Southwest England (a five-year undergraduate entry medical course consisting of approximately 1000 students) were invited to participate. Students across all year groups and backgrounds (international or home) were invited to achieve a diverse sample.

We undertook interviews face-to-face or online following Covid-19 safety guidelines. Interviews were semi-structured and mainly participant led, using Manzano’s [27] realist techniques. A pilot interview with a volunteer medical student from a different university refined this realist interviewing technique. Interviews followed one of three types: theory gleaning, refining or consolidating.

Theory gleaning interviews explored medical students’ perspectives. The initial programme theory was proposed to participants, and they articulated their understandings of if, how, why and under what conditions it applied to their real-world setting. Exemplar questions included: Are you or any of your peers planning to move abroad? What situations or circumstances [contexts/mechanisms] are important in enabling that [the outcome] to happen?

Theory refinement interviews then identified which themes applied to the participants most in their setting. Participants were asked how they understood the relationships within the developing programme theory alongside their own examples. Exemplar questions included: To what extent does the developing programme theory make sense to you? Which parts are most or least relevant to your setting? In your experience, what associations would you make?

Lastly, in theory consolidation interviews the near-final programme theory was presented to participants with a view to validating the connections between the contexts, mechanisms, and outcomes. Additionally, theory consolidation participants were invited to share their own recommendations based on the programme theory and discussion. Exemplar questions included: Which parts make the most sense to you and why? How does this CMOc play out in your setting? What recommendations would you make based on these findings? Ultimately, after the final interview, the final programme theory was produced.

Interviews were recorded and transcribed using Microsoft Word (version 16.58) software. Data was coded iteratively, using NVivo Release V1.6.1 (NVivo, QSR International Pty Ltd., Australia) software, and analysed identifying common themes and patterns. The frequency and conviction of connections between contexts, mechanisms and outcomes were used to develop CMOcs based on analysis of interview transcripts by the research team.

### Phase 3: Generating recommendations

The CMOcs identified formed the basis for a set of recommendations. Participants in theory consolidation interviews suggested recommendations to improve graduate retention. The authors refined the full list of these suggestions by grouping them into themes. Each theme was summarised into a singular final recommendation, ensuring it encompassed the relevant subpoints. Each final recommendation may relate to multiple CMOcs and were therefore not assigned to particular CMOcs. The final recommendations are intended to be generalisable that could positively influence the retention of domestic medical students as a whole.

### Reflexivity

Data was collected by EM, the lead author, who was a senior medical student intercalating between years 4 and 5 of medical school at the time. All authors made substantial contributions to the development of the programme theory. Although two of the co-authors were medical students (EM) or newly qualified doctors (DP), neither is intending to move abroad at the current time. The co-authors are KM who is a professor of medical education and DP a junior doctor, with KM and DP both having conducted and published realist research previously. Our team’s varied perspectives enhanced our insights and decision-making, ensuring differing viewpoints were considered.

## Results

### Phase 1: Generating the initial programme theory

**Figure 1:**
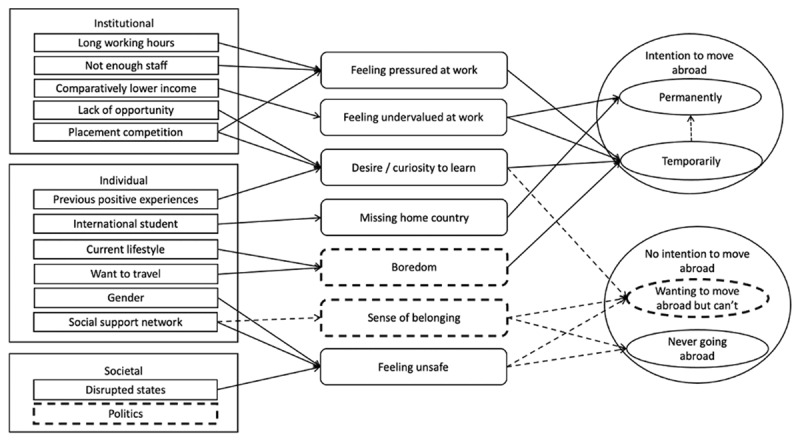
The initial programme theory developed by the rapid literature review. Contexts are represented by rectangles, mechanisms by rounded rectangles and outcomes by ovals. Solid arrows represent the linkages identified through the literature review and dashed arrows or outlines represent the linkages that arose through discussion by the authors.

The rapid review of literature generated potential contexts, mechanisms, and outcomes relating to medical students’ intentions towards moving abroad or not. These were collated into the initial programme theory (Figure 1), incorporating individual, institutional, and societal contexts.

### Phase 2: Realist interviews

Thirty realist interviews with medical students (Appendix 1, Table 2) were undertaken. All interviews took place before final year students’ elective (a six-to-eight-week period where final year medical students can choose to study abroad), with all but one participant having no prior experience of international healthcare environments. Good participant variance was achieved, although no third-year students volunteered to participate despite invitations being extended to all medical students. The interviews were evenly split across the three types of interviews: ten theory gleaning, ten theory refining and ten theory consolidating.

**Table 2:** The five context-mechanism-outcome configurations identified through analysis of our interview data with illustrative quotes.


CMOc	ILLUSTRATIVE QUOTE

CMOc 1. If medical students have not travelled to other countries and not experienced other cultures or medicine (C), they may move abroad once qualified (O) because they are excited to try something different (M1) or have a curiosity to learn outside of their current surroundings (M2).	Participant 22 ‘*I think wanting to experience a new place in the world is the main thing* [C1]*. I’d be excited to go somewhere new and see what it’s like in comparison* [M1] *and it would make me consider moving abroad* [O1].’ Participant 24 ‘*For me, they’ve* [Boston, United States] *got interesting neonatology research* [M2], *which is something I’m interested in* [C1], *so I would be really excited to go and explore* [O1].’

CMOc 2. If medical students have a strong social support network (friends and family) (C), they may feel they have a sense of belonging (M), which tends to lead to them staying where their social support network is after qualification (O).	Participant 4 ‘*I don’t think I’d ever move abroad* [O2] *because I love the UK and feel comfortable* [M4] *because my whole family is here* [C2].’

CMOc 3. If medical students have had previous positive experiences in other countries (C), then they may move abroad once qualified (O) because they are comfortable in moving countries (M).	Participant 15 ‘*Having lived in Italy for most of my life, I have loved this* [C3]. *It was very positive having experience of two countries … I enjoyed getting different perspectives and having different cultural influences in my life* [M5]. *So, I’d like to carry forward all those positive experiences* [O1].’

CMOc 4. If medical students perceive or have seen understaffing in the country they studied in (C), this could lead to them moving abroad once qualified (O), because of the potential to feel overwhelmed at work (M).	Participant 13 ‘*I think if our hospital is understaffed* [C4]… *I would see what my capacity is if I’m in that situation… But if it reaches a point where I can no longer do it* [M3] *then I would consider moving abroad* [O1].’

CMOc 5. If doctors are paid more in other countries (C), medical students may move abroad when qualified (O), because of the potential to feel undervalued at work (M).	Participant 1 ‘*I could move abroad* [O1] *and get paid a lot more* [C5]… *I feel like it does leave a sour taste in your mouth knowing that if you were in a different country, you would be getting paid more to do the same thing* [M6].’


Abbreviations: (C) depicts the context, (M) the mechanism and (O) the outcome.

**Figure 2:**
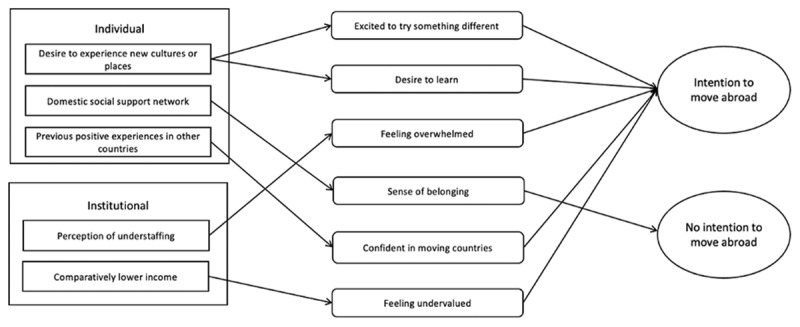
The final programme theory. Contexts are represented by rectangles, mechanisms by rounded rectangles and outcomes by ovals. The relevant significance of individual and institutional contexts and mechanisms, ascertained by data analysis, determined their placement order on the final programme theory. The most significant is at the top and least at the bottom of each domain. Contexts, mechanisms and outcomes are linked by solid arrows representing the context-mechanism-outcome configurations (CMOcs). Each CMOc consists of a starting context which connects to one or more mechanisms which connects to an outcome.

By analysing our interview data, we iteratively modified the initial programme theory to produce the final programme theory (Figure 2). During theory gleaning interviews, 17 additional contexts and 17 additional mechanisms were added to the initial programme theory. Research team discussion determined whether contexts, mechanisms and outcomes were distinct or should be combined with others. For example, ‘wanting to take a break’ and ‘feeling overwhelmed’, were combined into a single mechanism (feeling overwhelmed). We also decided to remove hypothetical scenarios, for example ‘wanting to move abroad but can’t’, to improve the real-world applicability of the resulting recommendations.

Contexts, mechanisms, and outcomes were refined using interview data. One challenging example was ‘long working hours’, which was removed late in the theory development, as interview participants argued that this is similar worldwide. Many contexts and mechanisms connecting to the outcome ‘no intention to move abroad’ were proposed by participants in theory gleaning interviews. However, through the development of the programme theory, less of these contexts and mechanisms were considered as important by the interview participants. Additional nuanced contexts, mechanisms and outcomes were discussed during the interview process, but the problem theory focusses on those with the most substantive and robust data, for clarity.

Appendix 2 shows the iterative development of the initial programme theory through to the final programme theory, showing further contexts, mechanisms and outcomes that were proposed and later removed as the process developed. The final programme theory (Figure 2) aims to explain the complex decision making behind medical students’ intentions towards moving abroad.

### Contexts

Five contexts were identified in the data as most important. These were C1) A want to experience new cultures or places, C2) Domestic social support network (including family and friends), C3) Previous positive experiences in other countries, C4) Perception of understaffing and C5) Underpaid on comparison. Demonstrative quotes for all contexts, mechanisms and outcomes identified as most important are included in appendix 3.

### Mechanisms

Six mechanisms were identified as most important. These were M1) Excited to try something different, M2) Desire or curiosity to learn, including medicine, cultures, or countries, M3) Feeling overwhelmed, including wanting to take a break, feeling pressured and feeling unsupported, M4) Sense of belonging, M5) Comfortable in moving countries, which was often based on personal experience in a specific country and M6) Feeling undervalued.

### Outcomes

Two outcomes remained after the interview process. These were O1) Intention to move abroad (temporarily or permanently) and O2) No intention to move abroad (in the country of study).

### Context-Mechanism-Outcome configurations

The final programme theory (Figure 2) illustrates the five CMOcs identified (Table 2). The interviews demonstrated many other potential connections between the contexts, mechanisms, and outcomes not included in our final programme theory (Appendix 2). Thus our final programme theory shows the connections with the most frequent and strongest supporting evidence based on our interview data. The CMOcs are demonstrated in Table 2, where sections correlating to the context, mechanism, and outcome are highlighted.

### Phase 3: Generating recommendations

#### Protected time within curricula for experiences of other healthcare systems

Medical students suggested that if they had sufficient opportunity to experience other health care systems in medical school or whilst working after qualification, they may not feel the need to move countries for longer periods.

‘*They had that experience*, [moving abroad] *they worked let’s say a few months and when they come back, they feel like they can stay and keep on working there.*’ (Participant 30)

‘*You don’t get an awful lot of experience… some medical schools are stopping students intercalating* [taking a year out of medicine completing a different degree] *outside of their institution, which gives even more difficulty… you can’t go and explore unless you’re doing it in your own time. I think increasing access to opportunities would be really good.*’ (Participant 24)

#### Increase the accessibility of postgraduate posts

Some participants felt that the competition for graduate and training posts indirectly contributes to the contexts and mechanisms on our final programme theory. Increasing accessibility of postgraduate posts, for example through increasing the number of posts overall, increasing numbers in certain specialties or increasing posts in different geographical locations allowing more choice and providing greater career certainty would reduce the desire to work abroad.

‘*There needs to be a move towards a more equitable management of student placement after graduation. Seeing people going through the FPO system* [two years post-graduate training] *having worked incredibly hard for five years and then feeling undervalued in the system isn’t fair.*’ (Participant 24)

#### Strengthen the domestic social support networks for graduates

When training places are limited, medical students explained how they felt those securing positions in locations where they do not know anyone may cause them to move abroad. Participants suggested that ensuring people are placed with others they know and greater emphasis on staff wellbeing would be beneficial.

‘*Focus more on staff well-being and social aspects to work. A social group doesn’t have to be anything external. Maybe something like 10 minutes in the morning.*’ (Participant 27)

‘*You want to be near your social support. I think encouraging this in work… I definitely know people that work on wards where they’re one really big family and I’ve never heard them saying they would go.*’ (Participant 28)

## Discussion

The aim of this realist evaluation was to better understand the interacting contexts and mechanisms that result in medical students wanting to move abroad or not. The realist interviews have developed our understanding of how medical students’ context influences their intention to move abroad or not and identified important mechanisms through the final programme theory.

The findings illustrate that the decision to move abroad is complex, but they have also identified important context-mechanism-outcome configurations. This realist evaluation extends former research by providing new insights leading to evidence-based recommendations that may be effective in persuading more medical students to remain in the country they trained in after graduation. The findings add to the body of evidence demonstrating the significance of context in healthcare professionals’ intentions on moving abroad after qualification.

Previous studies highlighted these contexts with specific focus on qualified doctors leaving the UK, citing the contexts of financial gain [9,10,11,12], understaffing [9,10,11,12,13], a wish to experience other cultures of places [9,10,11,13], and better training opportunities abroad [9,10,11]. Our study has found that medical students have similar opinions even before starting work. Gauld and Horsburgh’s study [11] of why UK doctors emigrate to New Zealand highlighted that younger doctors were more likely to want to move abroad due to quality-of-life considerations. Unsurprisingly our study of students uncovered many personal factors correlating with this, including a desire to experience new cultures or places and previous positive experiences in other countries.

Nonetheless, our study also found that medical students were considering institutional factors, such as financial gain and working conditions. We have identified that despite not having worked as doctors, medical students already have perceptions of what future working will be like and unfortunately this was often negative. It may be that medical students have this perception from their experiences on placements, through their own external work, negative influence by media, or discussions with qualified seniors.

There are fewer studies [9,12] that have begun to unearth the mechanisms connecting these contexts to outcomes, such as understaffing leading to feeling overwhelmed [12] and being underpaid on comparison leading to feeling undervalued [9,12]. This study identified that these mechanisms can also apply to medical students, as they may already hold perceptions in a similar way as their qualified counterparts. However, this study has identified further mechanisms and contexts, demonstrated in our final programme theory. Importantly, we have identified multiple mechanisms relating to individual contexts (excitement to try something different, a desire to learn, sense of belonging and confidence in moving countries) that were to the best of our knowledge previously not reported. Therefore this study deepens our understanding of how medical students may make these complex decisions, highlighting that it is often multifactorial, and thereby what strategies might persuade them to stay.

There has been study on the attitudes of medical students towards moving abroad outside the UK [16,17,18,19,20,21]. Despite being from developed [16,17,20,21] or developing countries [18,19] all of these studies identified the context of better training opportunities abroad. We identified a similar context in our study (placement competition leading to medical students wanting to leave) however it was removed after theory gleaning interviews as it was not considered the most relevant aspect. Our study highlights perceived comparatively lower pay as a context that may lead to an intention to move abroad. This was also identified as a context in both developed countries (Croatia [16,17], Spain [20] and Austria [21]) and developing countries (Ghana [19]). Our study has identified a possible mechanism for how this context may lead to medical students having an intention to move abroad (feeling undervalued). Our findings could therefore be applied in other countries (including those with different socio-economic backgrounds) that have identified this context within their population to identify and implement effective interventions.

Our final programme theory reflects our study population including the international students who participated. We acknowledge that international students may have different perspectives on moving abroad but the reasons why international students do not renew their visas is not currently clear in literature. This study has unearthed some mechanisms that specifically apply to international students (being confident in moving countries and missing a home country) (as seen in the programme theory’s development in Appendix 2) although these did not remain on the final programme theory as they were not the most important mechanisms to the whole study population. It does however highlight that this could be a population sub-group for future study.

Perhaps most significantly, the study demonstrates that medical students begin to form opinions about emigrating prior to graduation, even at the start of medical school. This would suggest that many of the current government initiatives to improve the retention of domestically trained doctors may be too little, too late. Based on the data, it seems an adjunctive approach to tackling staff shortages that is implemented in medical schools may be necessary, requiring greater co-operation with training programmes and regulators.

### Strengths and Limitations

There are a number of strengths of this study. It has demonstrated that, even prior to qualification, medical students can provide rich accounts of their thinking about moving abroad. By interviewing a diverse range medical students we successfully collated a range of viewpoints. Our inclusion of medical students from year groups spanning the start to the end of medical school has highlighted that potential solutions could be implemented at an early stage during medical studies. By using Manzano’s [27] realist interview techniques and RAMESES II guidelines [24] we have produced robust recommendations that may be transferable to other institutional settings.

However, it is important to acknowledge this study’s limitations. The contexts, mechanisms and outcomes, and CMOcs, are those deemed most important and relevant to our participants but are not exhaustive. As this study was undertaken in one institution in the UK, our final programme theory and recommendations may need tailoring for other settings or other countries. Our programme theory also details intentions, not observed outcomes. For example, some participants said they will move abroad, but we do not know if or when this will occur in reality. However, we argue that without intentions, outcomes will not occur. In addition, some contexts, and mechanisms, for example feeling overwhelmed, are perceptions based on involvement within healthcare settings as students on placement. We appreciate that some participants may already have other healthcare jobs, but perceptions may change once they qualify and work as doctors.

### Future research and practice implications

Our recommendations of protecting time within medical curricula to experience other healthcare systems, increasing accessibility of postgraduate posts to provide career certainty at home and strengthening UK-based social support networks for graduates could be applied in different institutions to help retain medical students after qualification. Future research could involve stakeholder evaluation of these recommendations and their implementation, perhaps in different institutional or professional contexts, or in different countries.

## Data Accessibility Statement

The data that support the findings of this study are available from the corresponding author upon request.

## Additional File

The additional file for this article can be found as follows:

10.5334/pme.1170.s1 supplementary File 1. Appendix 1–Appendix 3.

## References

[1] Humphries N, Crowe S, Brugha R. Failing to retain a new generation of doctors: qualitative insights from a high-income country. BMC Health Serv Res. 2018; 18: 144. DOI: 10.1186/s12913-018-2927-y 29486756 PMC5830046

[2] Joarder T, Rawal LB, Ahmed SM, Uddin A, Evans TG. Retaining Doctors in Rural Bangladesh: A Policy Analysis. International journal of health policy and management. 2018; 7(9): 847–858. DOI: 10.15171/ijhpm.2018.37 30316233 PMC6186485

[3] Aluttis C, Bishaw T, Frank MW. The workforce for health in a globalized context-glocal shortages and international migration. Glob Health Action. 2014; 13(7): 23611. DOI: 10.3402/gha.v7.23611 PMC3926986 24560265

[4] NHS Digital. NHS Vacancy Statistics England April 2015 – June 2022 Experimental Statistics. https://digital.nhs.uk/data-and-information/publications/statistical/nhs-vacancies-survey/april-2015---june-2022-experimental-statistics (accessed 14 January 2023).

[5] Bazouki X, Kalampokis N, Papoudou-Bai A, Bazoukis G, Grivas N. The increasing incidence of immigration and information-seeking behaviour of medical doctors in north-western Greece. Rural Remote Health. 2020; 20(1): 4877. DOI: 10.22605/RRH4877 32200643

[6] BMA. Staffing crisis in NHS laid bare, as new BMA analysis shows that three quarters of medical specialities face shortage of doctors. https://www.bma.org.uk/news/media-centre/press-releases/2017/september/staffing-crisis-in-nhs-laid-bare. (accessed 5 October 2021).

[7] Harris JR, Todaro MP. Migration, Unemployment and Development: A Two-Sector Analysis. The American Economic Review. 1970; 60(1): 126–142.

[8] Holland JL. Making vocational choices: a theory of vocational personalities and work environments. Englewood Cliffs, NJ: Prentice-Hall; 1985.

[9] Brennan N, Langdon N, Bryce M, et al. Drivers of international migration of doctors to and from the UK. https://www.gmc-uk.org/-/media/documents/drivers-of-international-migration-research-final-report_pdf-88769526.pdf (accessed 22 April 2022).

[10] Lambert TW, Smith F, Goldacre MJ. Why doctors consider leaving UK medicine: qualitative analysis of comments from questionnaire surveys three years after graduation. J R Soc Med. 2018; 111(1): 18–30. DOI: 10.1177/0141076817738502 29035667 PMC5784487

[11] Gauld R, Horsburgh S. What motivates doctors to leave the UK NHS for a ‘life in the sun’ in New Zealand; and, once there, why don’t they stay? Human Resoures for Health. 2015; 13(75). DOI: 10.1186/s12960-015-0069-4 PMC4563843 26350706

[12] Smith SE, Tallentire VR, Pope LM, Laidlaw AH, Morrison J. Foundation Year 2 doctors’ reasons for leaving UK medicine: an in-depth analysis of decision-making using semistructured interviews. BMJ Open. 2018; 8: 19456. DOI: 10.1136/bmjopen-2017-019456 PMC5855199 29500208

[13] Moss PJ, Lambert TW, Goldacre MJ, Lee P. Reasons for considering leaving UK medicine: questionnaire study of junior doctors’ comments. BMJ. 2004; 329(7477): 1263. DOI: 10.1136/bmj.38247.594769.AE 15469947 PMC534439

[14] Yang Y, Li J, Wu X, et al. Factors influencing subspecialty choice among medical students: a systematic review and meta-analysis. BMJ Open. 2019; 9: e022097. DOI: 10.1136/bmjopen-2018-022097 PMC6429728 30850399

[15] General Medical Council. The state of medical education and practice in the UK. https://www.gmc-uk.org/-/media/documents/somep-2020_pdf-84684244.pdf?la=en&hash=F68243A899E21859AB1D31866CC54A0119E60291 (accessed 6 October 2021).

[16] Kolčić I, Cikeš M, Boban K, et al. Emigration-related attitudes of the final year medical students in Croatia: a cross-sectional study at the dawn of the EU accession. Croatian Medical Journal. 2014; 55(5): 452–8. DOI: 10.3325/cmj.2014.55.452 25358878 PMC4228300

[17] Bojanic A, Bojanic K, Likic R. Brain drain: final year medical students’ intentions of training abroad. Postgraduate Medical Journal. 2015; 91(1076): 315–21. DOI: 10.1136/postgradmedj-2014-132908 25995369

[18] Kizito S, Mukunya D, Nakitende J, Nambasa S, et al. Career intentions of final year medical students in Uganda after graduating: the burden of brain drain. BMC Medical Education. 2015; 15(1): 122. DOI: 10.1186/s12909-015-0396-0 26231749 PMC4522140

[19] Lassey AT, Lassey PD, Boamah M. Career destinations of University of Ghana Medical School graduates of various year groups. Ghana Medical Journal. 2013; 47(2): 87–91. 23966746 PMC3743114

[20] Bernardini-Zambrini D, Barengo N, Bardach A, Hanna M, Núñez JM. Emigrate or not? How would the next Spanish generation of physicians decide? A study on emigration-related reasons and motivations of advanced medical students in 11 Universities in Spain. Aten Primaria. 2011; 43(5): 222–6. DOI: 10.1016/j.aprim.2010.01.017 20416980 PMC7024900

[21] Scharer S, Freitag A. Physicians’ exodus: why medical graduates leave Austria or do not work in clinical practice. Wien Klin Wochenschr. 2015; 127(9–10): 323–9. DOI: 10.1007/s00508-015-0786-7 25931135

[22] Pawson R, Tilley N. Realistic evaluation. London: SAGE; 1997.

[23] Emmel N, Greenhalgh J, Manzano A, Monaghan M, Dalkin S. Doing realist research. London: SAGE; 2018. DOI: 10.4135/9781526451729

[24] Wong G, Westhorp G, Manzano A, Greenhalgh J, Jagosh J, Greenhalgh T. RAMESES II reporting standards for realist evaluations. BMC Med. 2016; 14(1): 96. DOI: 10.1186/s12916-016-0643-1 27342217 PMC4920991

[25] Pawson R. Theorizing the interview. The British Journal of Sociology. 1996; 47(2): 295. DOI: 10.2307/591728

[26] Pawson R, Tilley N. Realistic evaluation. http://www.communitymatters.com.au/RE_chapter.pdf. (accessed 15 October 2021).

[27] Manzano A. The craft of interviewing in realist evaluation. Evaluation. 2016; 22(3): 342–360. DOI: 10.1177/1356389016638615

[28] Marchal B, Van Belle S, Van Olmen J. Is realist evaluation keeping its promise? A review of published empirical studies in the field of health systems research. Evaluation. 2012; 18(2): 192–212. DOI: 10.1177/1356389012442444

[29] Greenhalgh J, Manzano A. Understanding ‘context’ in realist evaluation and synthesis. International Journal of Social Research Methodology. 2021. DOI: 10.1080/13645579.2021.1918484

[30] Coldwell M. Reconsidering context: Six underlying features of context to improve learning from evaluation. Evaluation. 2019; 25(1): 99–117. DOI: 10.1177/1356389018803234

[31] Grant MJ, Booth A. A typology of reviews: an analysis of 14 review types and associated methodologies. Health Information & Libraries Journal. 2009; 26: 91–108. DOI: 10.1111/j.1471-1842.2009.00848.x 19490148

