# Supplementary material for: Medical Student Intentions to Move Abroad: A UK-Based Realist Evaluation
Source: Perspect Med Educ. 2024 Feb 21;13(1):141–50. doi: 10.5334/pme.1170 (PMC10885844; doi:10.5334/pme.1170)
Supplement: supplementary File 1. — Appendix 1–Appendix 3. [file pme-13-1-1170-s1.pdf]

Appendix 1:

Table 1: Search terms used to generate the initial programme theory

**Population**	**Outcome**	**Contexts**
Medic* student*	Mov* Abroad	Work* condition*
International medic* student*	Emigrat*	Underpaid
Doctors[§]	Migrat*	Learn* opportunity
	Travel*	Lifestyle
		Gender

*These words were truncated, as such the database would generate results for the word written with any ending.

[§]'Doctors' was also searched for. There is vast literature exploring doctors' perspectives and it is sensible to suggest that they may have similar opinions to medical students, as they would have been a medical student themselves, and in some cases recently. It is also noted that medical students are unlikely to be able to move abroad during training, which likely accounts for the lack of substantial literature with a specific focus on medical students. As this search was used to generate the initial programme theory, the realist interviews (Phase 2) edited these if medical students perceived them not to be relevant.

Table 2: Realist interview participant demographics.

**Demographic**	**Number of participants (percentage) (*n* = 30)**
Gender	
Male	12 (40%)
Female	18 (60%)
Year of Medical School	
1	4 (13%)
2	8 (27%)
3	0 (0%)
4	6 (20%)
5	12 (40%)
International student	
Yes	5 (17%)
No	25 (83%)
Previous higher education experience	
Yes	1 (3%)
No	29 (97%)

Appendix 2: A collection of figures illustrating the programme theory's development.

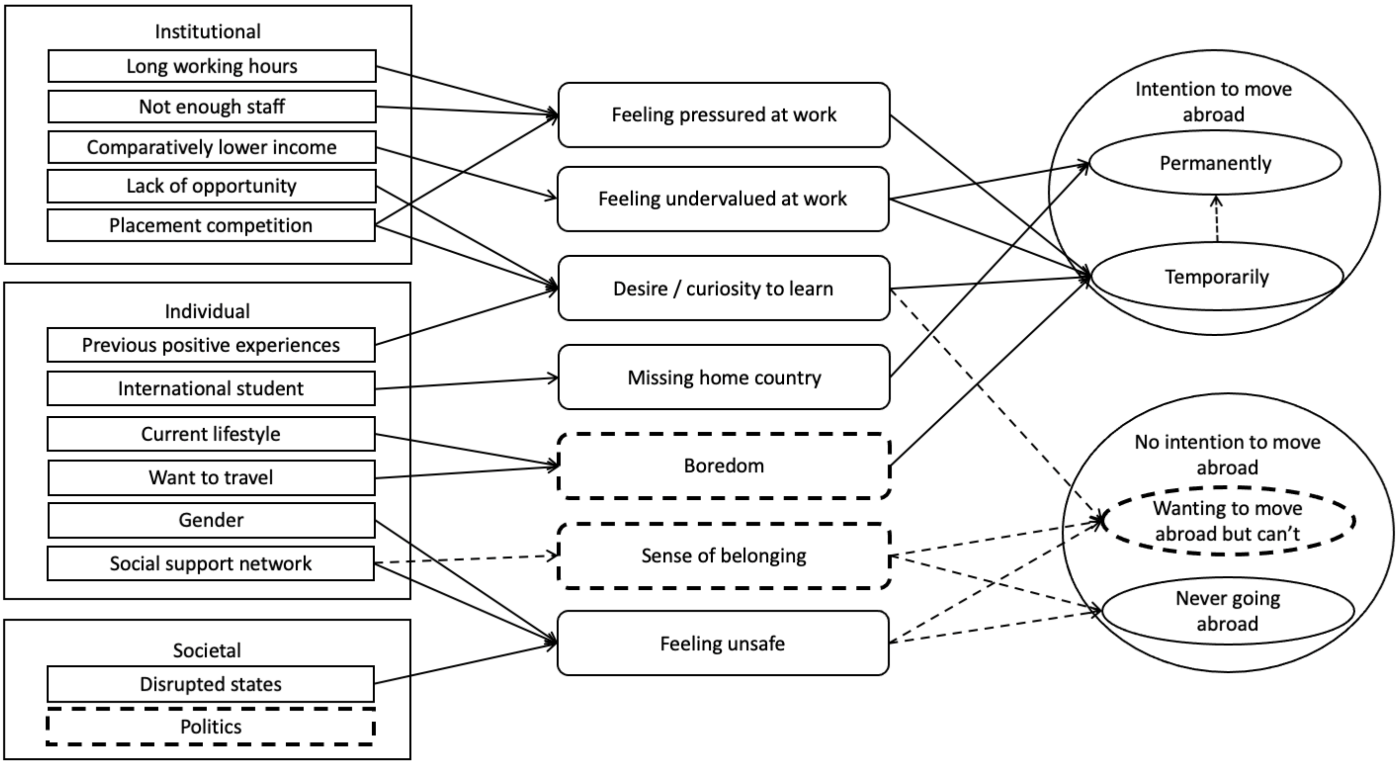

2. Programme Theory after Theory Gleaning Interviews

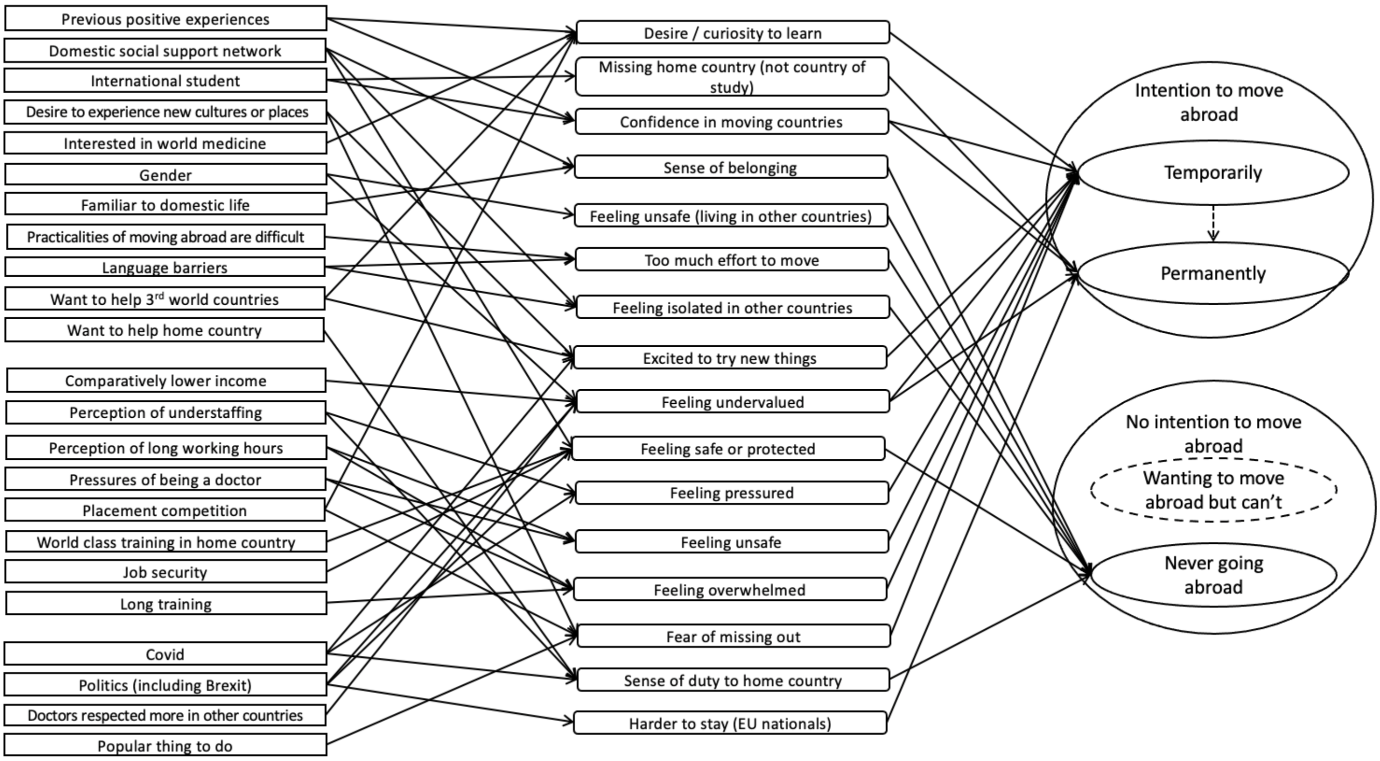

3. Programme Theory after Theory Refining Interviews

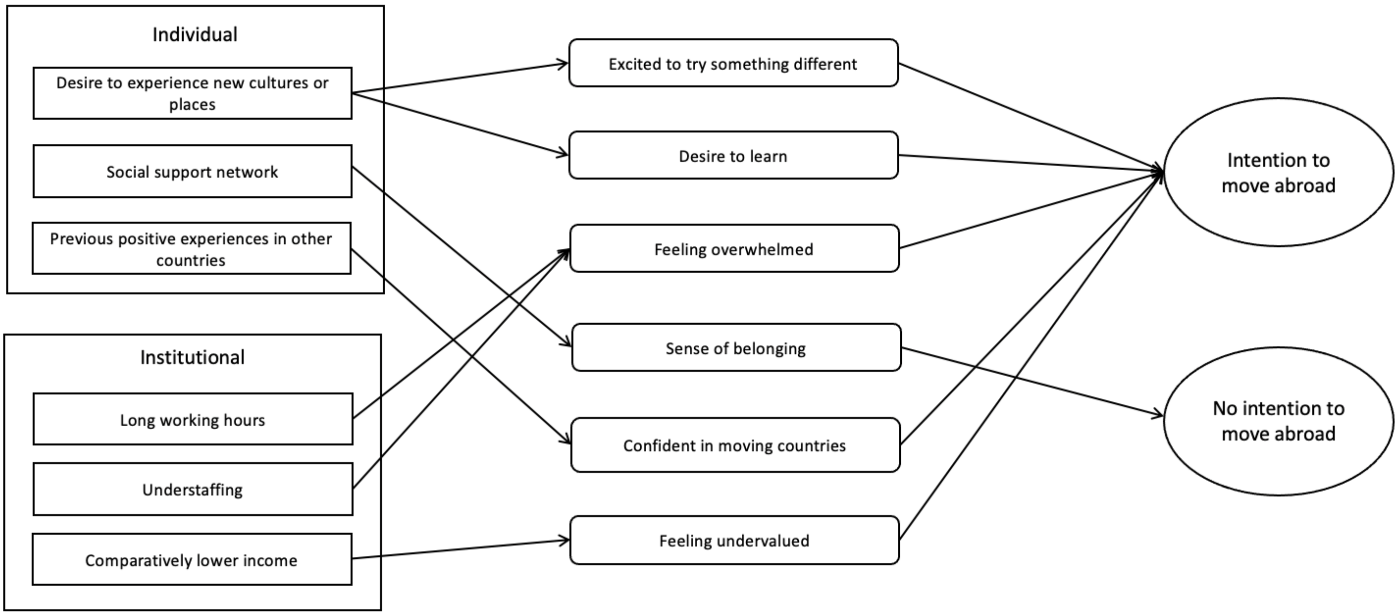

4. Final Programme Theory, after theory consolidation interviews.

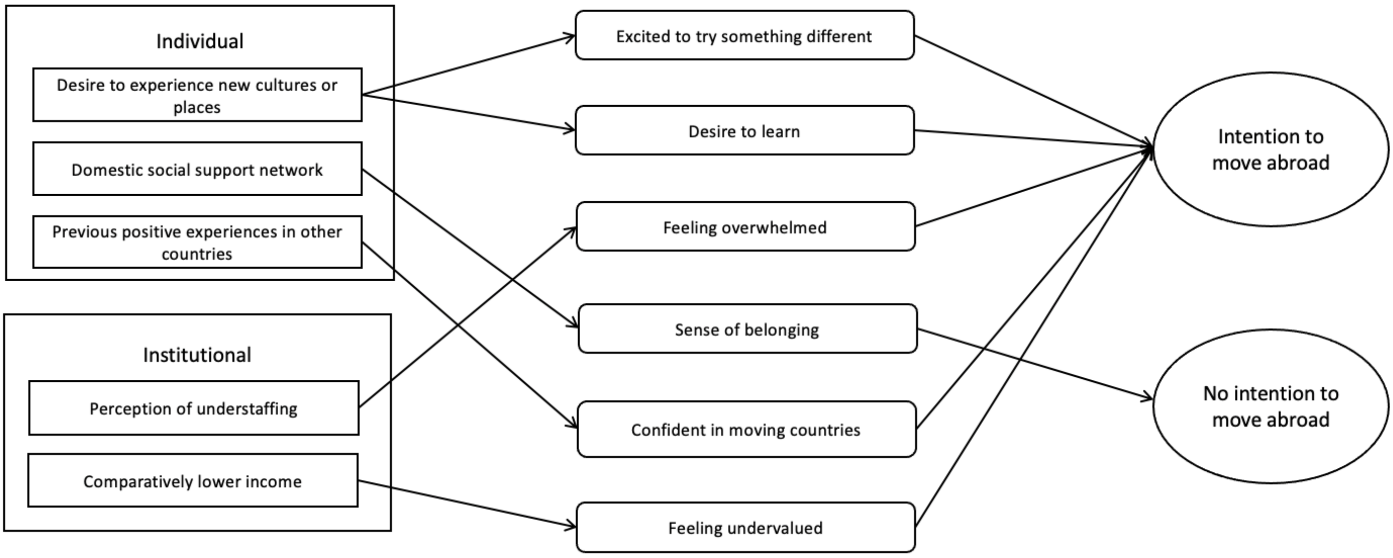

Appendix 3: Exemplar quotes demonstrating contexts mechanisms and outcomes.

C1) A want to experience new cultures or places

"*The main reason* [I would move abroad] *is so I could experience living in a different country anyway… I haven't experienced going to many other countries apart from in the EU* [European Union] *and very touristy parts, which obviously isn't the same experience as living in a place… working as a doctor if I'm able to, why not?"* (Participant 3)

C2) Domestic social support network (including family and friends)

"*Social network, your friends or family, that would be the main thing that would stop me or people I've spoken to from moving to another country, if they didn't want to leave our family and friends behind."* (Participant 29)

C3) Previous positive experiences in other countries

"*So it's been a positive experience for me moving from home* [Outside the UK] *alone independently from my family, coming here* [UK]*… there's that multicultural aspect to me that just wants to try out more different countries."* (Participant 13)

C4) Perception of understaffing

"*There's not enough team around them* [doctors] *or they don't feel supported… I could understand why they might feel tempted to leave. I think this will be a strong reason to move permanently."* (Participant 15)

C5) Underpaid on comparison

"Whenever people talk about leaving the UK, the main focus is the fact that they're underpaid and having to do more work." (Participant 25)

M1) Excited to try something different

“I'm excited to learn about other places and how it is to live in a different country. Every time you move, it's kind of like a fresh start.” (Participant 7)

M2) Desire or curiosity to learn, including medicine, cultures, or countries

“I think experiencing a different healthcare system would be cool because there's differences between all of them. When I was doing my SSU [special study unit, a student selected component of the medical course] *we looked at the Panama healthcare system which was cool and got me interested in doing that sort of thing.”* (Participant 12)

M3) Feeling overwhelmed, including wanting to take a break, feeling pressured and feeling unsupported

“I could be working as an F2 [doctor in their second year after qualifying] *in the NHS and feeling like I'm not making a difference and I'm exhausted and still struggling to balance my work life. Yeah, I think if that's the case, I definitely would look at working abroad.”* (Participant 1)

M4) Sense of belonging

“I think when you grow up in a country, you tend to have a sense that you belong there or you kind of feel like this where your home is and so moving away from that would be challenging.” (Participant 2)

M5) Comfortable in moving countries, which was often based on personal experience in a specific country

“I think he [friend, medical student] *moved around a lot, he was in New Zealand, he's now in Vienna, I think that people who have lived abroad are more willing or more interested by the idea of working abroad because they're used to moving around”* (Participant 5)

M6) Feeling undervalued

“It's [being underpaid on comparison] *quite demotivating or you feel quite undervalued within the whole institution, which feels like it can only be rectified if you move out.”* (Participant 25)

O1) Intention to move abroad (temporarily or permanently)

“I'm definitely planning on doing a foundation year three [an extra year before starting speciality training] *abroad in Australia or New Zealand.”* (Participant 8)

“*It's always been sort of a decision for me. I knew I wasn't going to stay in the UK when I came to medical school.”* (Participant 7)

O2) No intention to move abroad (in the country of study)

“I don't think I'd ever move abroad because I love the UK and I feel comfortable because my whole family is here.” (Participant 4)
